# Bedside assistant models in robotic abdominal and pelvic surgery: a systematic review of operative efficiency, training exposure, and perioperative safety

**DOI:** 10.1007/s11701-026-03810-x

**Published:** 2026-08-11

**Authors:** Rathin Gosavi, Baxter Smith, Chun Yin Yan, Amrish Rajkomar, Satish Warrier

**Affiliations:** 1 Department of Surgery, School of Clinical Sciences at Monash Health), Monash University, Melbourne, VIC Australia; 2 Department of Colorectal Surgery, Monash Health, Melbourne, VIC Australia; 3 Department of Colorectal Surgery, Cabrini Health, Melbourne, VIC Australia; 4 Department of Colorectal Surgery, Alfred Health, Melbourne, VIC Australia; 5 General Surgery and Gastrointestinal Clinical Institute, Epworth Healthcare, Melbourne, VIC Australia

**Keywords:** Bedside assistant, Robotic surgery, Abdominal surgery

## Abstract

Robotic surgery depends on effective bedside assistance, yet staffing models vary between physician and non-physician assistants. We systematically reviewed comparative evidence evaluating these models in robotic abdominal and pelvic surgery. A PRISMA compliant systematic review was performed. PubMed, Embase, Ovid MEDLINE, Cochrane CENTRAL/CCTR and ClinicalTrials.gov were searched from inception to 12 April 2026. Comparative adult clinical studies were eligible if they directly compared physician bedside assistance with non-physician bedside assistance during robot-assisted abdominal or pelvic surgery. The primary outcome domain was operative efficiency. Training exposure and perioperative safety were secondary domains. Risk of bias was assessed using ROBINS-I. Meta-analysis was not performed because of clinical and outcome heterogeneity. The search identified 1587 records, with 4 additional records from other sources. After duplicate removal, 693 records were screened and 22 full texts assessed; 3 observational studies met inclusion criteria. These included colorectal, gynaecologic oncology, and transplant/hepatopancreatobiliary robotic settings. Across studies, non-physician or dedicated bedside assistance was associated with favourable time-based efficiency outcomes, including shorter operative or console time in selected procedures and improved operational metrics in a dedicated team model. Training exposure was inconsistently reported, although one programme-level study found increased fellow primary surgeon participation after implementation of a dedicated robotic team. Safety outcomes did not show an obvious harm signal, but reporting was heterogeneous and studies were subject to confounding. Direct comparative evidence evaluating physician versus non-physician bedside assistant models in robotic abdominal and pelvic surgery is limited. Available data suggest possible efficiency benefits without an evident safety penalty, but stronger prospective studies with standardised efficiency, training and safety outcomes are needed.

**Supplementary Information:**

The online version contains supplementary material available at 10.1007/s11701-026-03810-x.

## Introduction

Robot-assisted surgery has reshaped abdominal and pelvic operative practice, but it has also altered intraoperative team dynamics. By physically separating the primary surgeon at the console from the patient, robotic platforms increase reliance on a skilled bedside assistant to maintain exposure, exchange instruments, provide suction and retraction, facilitate stapling and specimen handling, troubleshoot equipment issues, and coordinate emergency undocking when required [1, 2]. Although many of these tasks are also present in laparoscopic surgery, robotics accentuates their importance because the console surgeon cannot directly intervene at the bedside, and workflow interruptions may accumulate over complex procedures.

Bedside staffing models vary between institutions. In some programmes, bedside assistance is provided by medical practitioners, most commonly trainees and occasionally a second consultant surgeon. In others, non-physician assistants, including physician assistants, nurse practitioners, registered nurse first assistants, and surgical care practitioners, provide first-assist support as dedicated or high-volume members of the robotic team [1, 3, 4]. These models differ not only by credentialing, but also by stability, familiarity with robotic equipment and room setup, and the extent to which bedside roles are standardised. In practice, the “assistant model” often reflects a broader service structure: who assists, how consistently they assist, and how embedded they are within the robotic workflow.

These staffing decisions sit at the intersection of workforce sustainability and surgical education. Reliance on trainees for bedside assistance may compete with ward cover, perioperative responsibilities, and opportunities for console operating [5, 6]. Conversely, bedside participation provides important exposure to instrument management, troubleshooting, communication, and emergency response during robotic procedures. Any redesign of bedside staffing therefore requires balancing operative efficiency with training opportunity, while preserving the bedside competencies needed for safe independent robotic practice.

These issues are particularly relevant to colorectal surgery. Robotic pelvic dissection and stapling-heavy workflows depend on coordinated bedside assistance, including suction and exposure, stapling support, specimen extraction, and management of technical interruptions. Rectal resections are often prolonged and interruption-prone, and a stable bedside assistant may improve operative flow by anticipating key steps and reducing non-console delays. At the same time, colorectal programmes face increasing pressure to deliver efficient robotic lists while expanding trainee console exposure [7, 8].

Despite the growing use of non-physician first-assist roles in robotic surgery, comparative outcome data remain limited. Much of the literature describes bedside assistant competencies, learning curves, or training curricula rather than evaluating staffing models as service interventions [2, 9–11]. Where outcomes are reported, they most commonly relate to operative efficiency, including console time, operative time, and overall case duration, while training exposure and safety endpoints are less consistently captured. We therefore undertook a systematic review of comparative clinical studies evaluating physician versus non-physician bedside assistant models in robotic abdominal and pelvic surgery, focusing on operative efficiency, trainee console exposure, and perioperative safety.

## Methods

### Protocol and reporting

This systematic review was performed in accordance with the PRISMA 2020 statement. A protocol was developed a priori and registered with PROSPERO (CRD420261431706). A structured narrative synthesis was planned because of anticipated clinical heterogeneity across procedures and specialties, variable definitions of operative efficiency outcomes, and the expected small number of eligible studies directly comparing bedside assistant models.

### Eligibility criteria

We included comparative clinical studies involving adults (≥ 18 years) undergoing robot-assisted abdominal or pelvic surgery. Studies were eligible if they directly compared a medical practitioner bedside assistant model (resident/registrar, fellow, or attending/consultant surgeon assisting at bedside) with a non-physician bedside assistant model, including physician assistants, nurse practitioners, registered nurse first assistants, surgical care practitioners, or employed surgical assistants. Retrospective and prospective cohort studies and quasi-experimental before-and-after studies were eligible. Paediatric-only cohorts, simulation-only studies without clinical cases, single-arm descriptive series without a relevant comparator, editorials, commentaries, and narrative reviews were excluded.

### Outcomes

The primary outcome domain was operative efficiency. Because time-based endpoints were reported variably, outcomes were extracted using a prespecified hierarchy: console time, operative or procedure time, and operating room time or case length. Where available, component intervals such as docking time, turnover time, or non-console time were extracted descriptively. Secondary outcomes were training exposure, including trainee console time, proportion of case performed by trainees, or trainee participation as primary surgeon, and perioperative safety outcomes, including conversion to open surgery, estimated blood loss, transfusion, intraoperative adverse events, postoperative complications, length of stay, readmission, reoperation, and mortality.

### Information sources and search strategy

We searched PubMed, Embase, Ovid MEDLINE including Epub Ahead of Print, In-Process and Other Non-Indexed Citations, Cochrane CENTRAL/CCTR, and ClinicalTrials.gov from database inception to 12 April 2026. he search strategy combined terms for robot-assisted surgery with terms relating to bedside assistance and assistant roles, including “robotic”, “robot”, “anterior resection”, “assistance”, “second surgeon”, “assistant”, “assist” and “bedside”. Because terminology for non-physician bedside assistants varies across health systems and specialties, a supplementary targeted search was also performed using role-specific terms including “physician assistant”, “physician associate”, “PA”, “nurse practitioner”, “advanced nurse practitioner”, “NP”, “registered nurse first assistant”, “RNFA”, “surgical care practitioner”, “SCP”, “surgical first assistant”, “advanced practice provider”, “APP”, “employed surgical assist” and “non-physician assistant”, combined with robotic surgery terms. No outcome filters were applied, and searches were not restricted by surgical specialty. Full database-specific search strategies are provided in Supplementary Table 1. Reference lists of included studies and relevant reviews were screened manually to identify additional eligible records.

### Study selection

Titles and abstracts were independently screened by two reviewers against prespecified eligibility criteria. Full texts of potentially eligible studies were retrieved and independently assessed by two reviewers. Disagreements were resolved by consensus, with third reviewer adjudication if required. Reasons for exclusion at full-text review were recorded and are presented in the PRISMA flow diagram. The supplementary targeted search using role-specific non-physician assistant terminology did not identify any additional eligible studies.

### Data extraction

Two reviewers independently extracted data using a piloted extraction form. Extracted items included study design, country, setting, specialty, procedure type, sample size, patient selection criteria, definitions of physician and non-physician bedside assistance, and whether the staffing model reflected a stable or dedicated bedside team. For outcomes, we extracted the definition used, units of measurement, summary statistics, effect estimates, and measures of variance where available. Where adjusted analyses were reported, covariates included in the models were recorded.

### Risk of bias assessment

Risk of bias was assessed using ROBINS-I for non-randomised comparative studies. Domains included confounding, participant selection, classification of interventions, deviations from intended interventions, missing data, outcome measurement, and selection of reported results. Assessments were performed independently by two reviewers and resolved by consensus. Overall risk of bias judgements were assigned according to ROBINS-I guidance.

### Synthesis methods

Meta-analysis was not performed because of heterogeneity in procedure type, assistant model implementation, and outcome definitions, and because only three eligible comparative studies were identified. Findings were synthesised narratively by outcome domain: operative efficiency, training exposure, and perioperative safety. Effect estimates, p values, and adjusted results were reported where available.

## Results

### Study selection

**Fig. 1:**
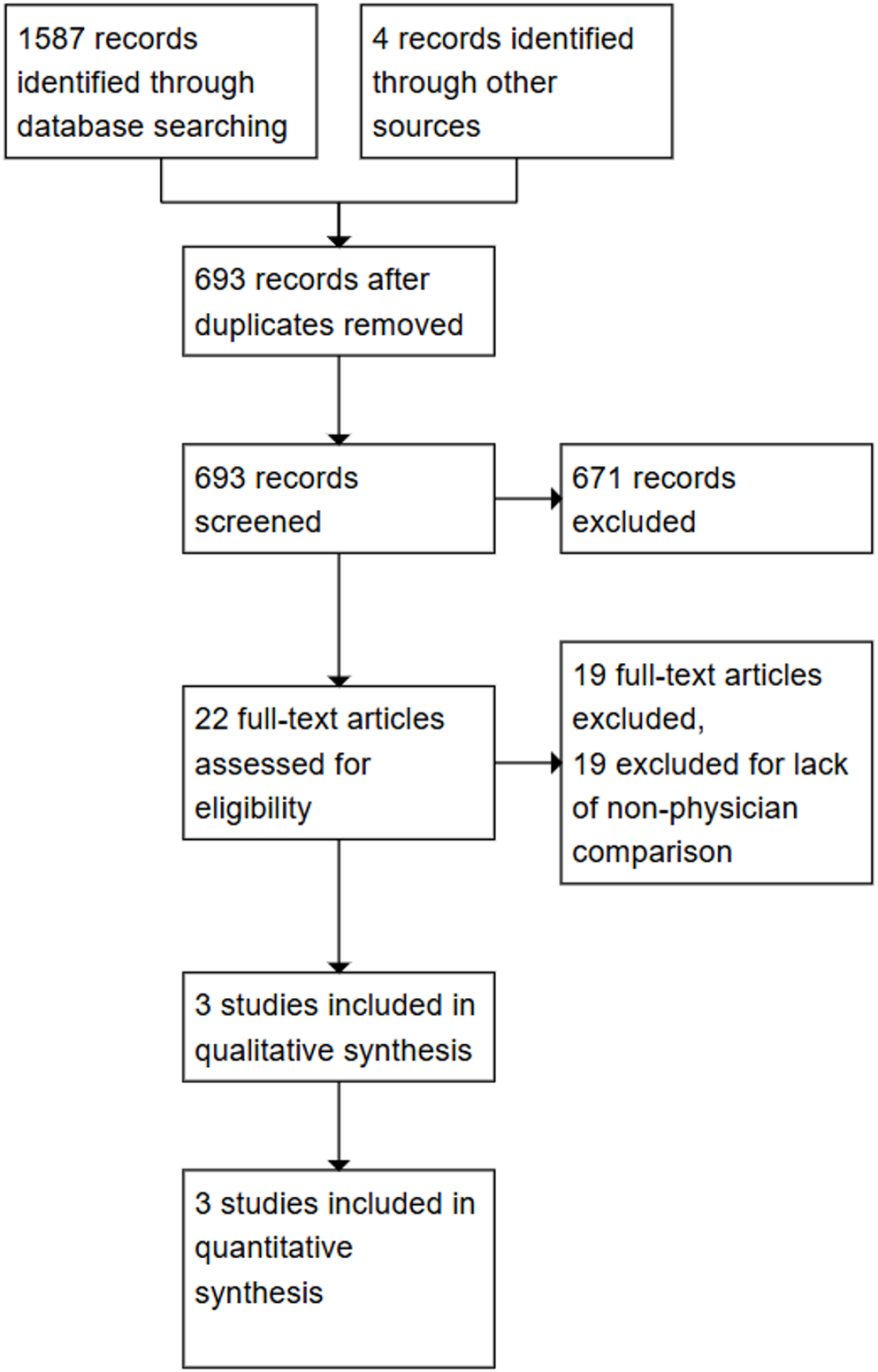
PRISMA flow diagram

The search identified 1587 records through database searching and 4 additional records through other sources. After duplicate removal, 693 records were screened by title and abstract. Twenty-two full-text articles were assessed for eligibility, of which 19 were excluded because they did not include a comparator arm with a non-physician bedside assistant model. Three studies met inclusion criteria and were included in the qualitative synthesis (Fig. 1).

### Study characteristics

Three observational comparative studies were included (Table 1): one colorectal cohort study, one gynaecologic oncology cohort study, and one programme-level cohort evaluating implementation of a dedicated robotic transplant operating room team in robotic transplant and hepatopancreatobiliary surgery. Across studies, non-physician bedside assistance included employed surgical assists, physician assistants, and/or registered nurse first assistants, while physician bedside assistance comprised trainees, attending surgeons, or second surgeons assisting at the bedside. The included studies differed in specialty, procedure type, assistant model implementation, and outcome definitions, precluding quantitative pooling.

**Table 1:** Characteristics of included studies

Study, year	Country	Setting	*N*=	Study design	Assistant type	1^o^ outcome	2^o^ outcomes
Hill, 2023	USA	HPB and transplant	402	Cohort	Physician vs. PA/RN	Fellow training cases	Safety, operative time, conversion rate, transfusion, complications, mortality
Leggett, 2022	USA	Gyne	1600	Cohort	Physician vs. PA/RN	Console time, EBL	
Oruc, 2026	USA	Colorectal	148	Cohort	Physician vs. PA	Console time	EBL, Cx, LOS, operative time

Abbreviations: *EBL* estimated blood loss, *HPB* hepatopancreatobiliary, *LOS* length of stay, *PA* physician assistant, *RN* registered nurse, *Cx* complications

### Operative efficiency outcomes

All three studies reported time-based efficiency outcomes, although the specific metrics varied.

In robotic colorectal surgery [9], dedicated bedside assistance by a physician assistant was associated with more favourable efficiency metrics in rectal resections. The study cohort included 148 robotic colorectal cases overall, comprising 90 rectal resections and 58 sigmoid resections; rectal-specific efficiency outcomes are therefore reported for the 90 rectal cases. Among 90 robotic rectal cases, dedicated bedside assistance was associated with shorter median operation time (213 vs. 255 min, *p* < 0.001) and shorter non-console time (92 vs. 118 min, *p* < 0.001), with a smaller reduction in active console time (98 vs. 117.5 min, *p* = 0.05) and a borderline difference in total console time (122 vs. 139 min, *p* = 0.06). In sigmoid resections, total operation time and total console time were similar between groups, but the dedicated assistance group demonstrated improved console utilisation metrics.

In gynaecologic oncology [10], employed surgical assist was associated with shorter adjusted console time and lower adjusted estimated blood loss compared with physician/second-surgeon assistance. After adjustment for case factors including BMI, uterine weight, fourth robotic arm use, benign versus malignant pathology, and surgeon-rated difficulty, employed assist was associated with a reduction in adjusted console time of 0.32 h (19.2 min; *p* < 0.001) and a reduction in adjusted estimated blood loss of 47.5 mL (*p* < 0.001).

In robotic transplant and hepatopancreatobiliary surgery [11], implementation of a dedicated robotic transplant operating room team was associated with improved operational metrics. Turnaround time decreased from 116 to 94 min (*p* = 0.001), and in-room-to-incision time decreased from 65 to 46 min (*p* = 0.0001).

### Training exposure outcomes

Training exposure was the least consistently reported outcome domain. Only one included study reported meaningful training exposure data, and these were programme-level participation metrics rather than direct measures of trainee console time, proportion of case performed, or autonomy progression. In the transplant/HPB programme evaluation [11], the presence of a trained robotic first assist increased from 17.2% to 95.1% (*p* < 0.0001) following implementation of the dedicated team model. Fellow involvement in any role increased from 58.7% to 76.4% (*p* < 0.0001), and when fellows were present, participation as primary surgeon increased from 18.9% to 94.2% (*p* < 0.0001). The colorectal and gynaecologic oncology studies did not report trainee console exposure outcomes in a comparable format.

### Perioperative safety outcomes

Safety outcomes were variably reported across the three included studies.

In the colorectal cohort [9], dedicated bedside assistance was not associated with higher perioperative morbidity. In rectal resections, there were no significant differences in estimated blood loss, intraoperative complications, early postoperative complications, or length of stay, and no anastomotic leaks were observed. In sigmoid resections, complication profiles were similarly comparable between groups.

In the gynaecologic oncology cohort [10], employed surgical assist was associated with lower adjusted estimated blood loss, as above. Other safety outcomes were not consistently reported in a form suitable for cross-study comparison.

In the transplant/HPB programme study [11], conversion to open operation in elective or semi-elective cases decreased from 9.5% to 3.8% (*p* = 0.04). No emergent conversions, robotic-specific complications, or 30-day deaths were reported in either era.

### Risk of bias

**Fig. 2:**
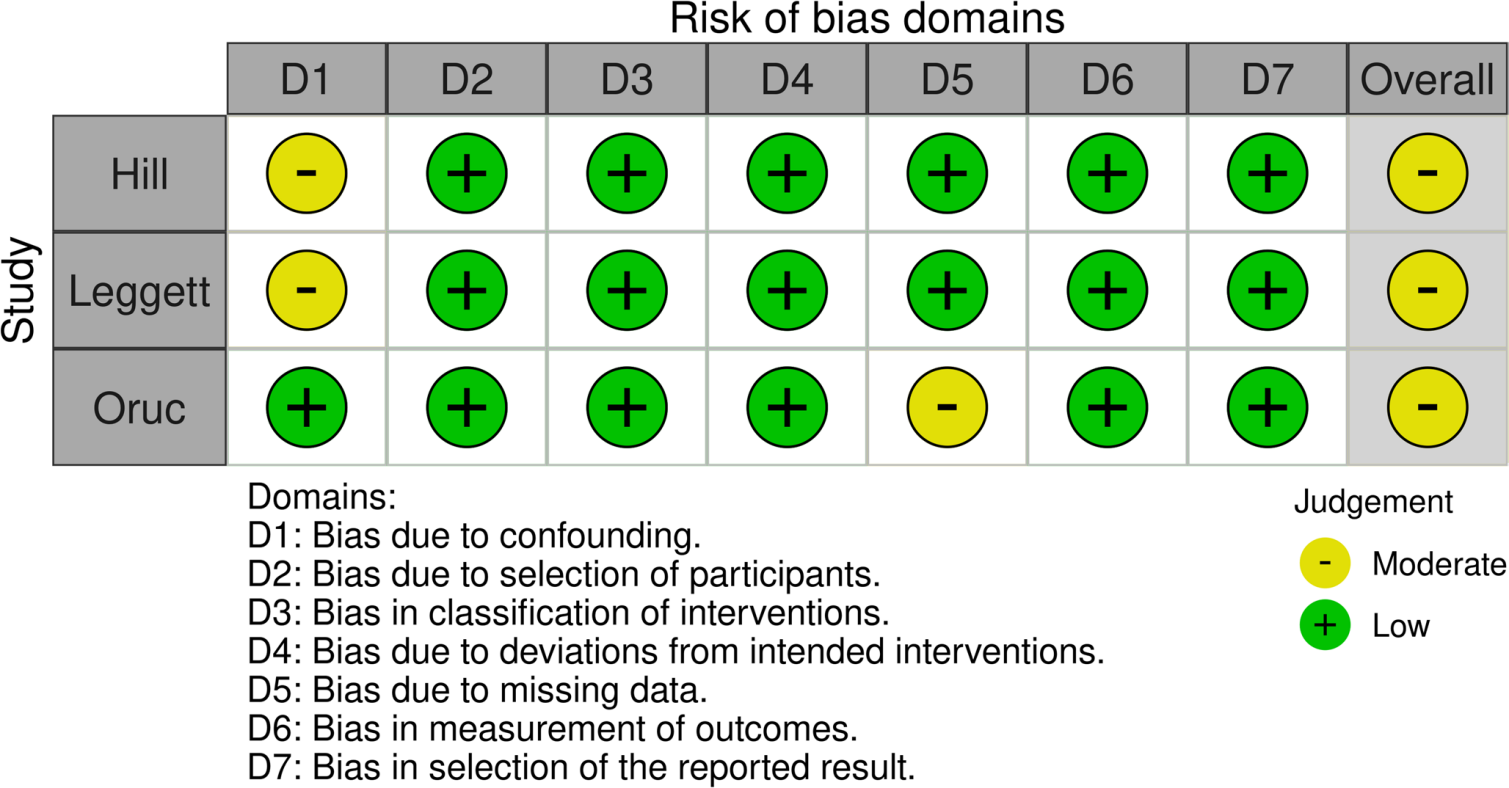
Risk of bias assessment

All three included studies were non-randomised and were judged to have moderate overall risk of bias using ROBINS-I (Fig. 2). The main concern was confounding due to non-random allocation of assistant models, case mix, surgeon preference and programme-era effects, particularly in the before-and-after programme-level study. However, assistant models were clearly classified, time-based outcomes were objectively measured, missing outcome data were not a major concern in the reported analyses, and there was no clear evidence of selective outcome reporting. These factors supported an overall moderate rather than serious risk-of-bias judgement. Training exposure and perioperative safety outcomes were less consistently defined and reported.

## Discussion

This systematic review identified only three comparative observational studies directly evaluating physician versus non-physician bedside assistant models in robotic abdominal and pelvic surgery [9–11]. Despite increasing use of non-physician first-assist roles in robotic programmes, comparative outcome data remain sparse and heterogeneous [1, 2]. Across the included studies, time-based efficiency endpoints were the most consistently measured outcomes and were the most likely to differ between assistant models, whereas training exposure and perioperative safety were reported less consistently, limiting inference in these domains.

Two of the three included studies suggested an association between non-physician bedside assistance and more favourable operative efficiency. In robotic colorectal surgery, dedicated bedside assistance by a physician assistant was associated with shorter operation time and shorter non-console time for rectal resections, with smaller differences in console time measures [9]. In sigmoid resections, overall operation and console times were similar, but console utilisation metrics improved, consistent with fewer interruptions rather than shorter total case duration. These observations are clinically plausible. Rectal resections are prolonged and interruption-prone, with multiple phases requiring coordinated bedside support, including suction and exposure, stapling preparation, specimen extraction, and troubleshooting [1, 12]. A stable bedside assistant who anticipates workflow and resolves technical interruptions promptly may reduce non-console inefficiencies, particularly in complex pelvic cases. In gynaecologic oncology, employed surgical assist was associated with shorter adjusted console time and lower adjusted estimated blood loss compared with physician/second-surgeon assistance after case-mix adjustment [10]. However, both studies were observational, and it remains unclear whether the observed associations reflect assistant credential, stability of the bedside role, assistant experience, surgeon preference, or broader programme maturity. The transplant/HPB programme evaluation did not isolate bedside assistant credential in the same way, but showed more favourable operational metrics after implementation of a dedicated team model [11], supporting the broader concept that standardisation and stability within robotic theatre teams may influence efficiency outcomes.

Training exposure should be considered an important but under-evidenced outcome domain in the current literature. Only the transplant/HPB programme study reported trainee participation metrics, showing a marked increase in fellows acting as primary surgeon after introduction of a dedicated team model alongside increased trained first-assist coverage. This suggests that structured bedside staffing can coexist with increased trainee operating, but does not capture trainee console minutes, autonomy progression, or the proportion of each case performed [5, 6, 13]. Its generalisability to colorectal training pathways is also uncertain. Future evaluations should include standardised training metrics, including trainee console time, proportion of case performed, dual-console utilisation, and objective autonomy or performance assessments. Without such data, the effect of bedside staffing redesign on robotic training remains incompletely evidenced.

Perioperative safety outcomes did not demonstrate an obvious harm signal associated with non-physician bedside assistance. The colorectal study reported no increase in perioperative adverse outcomes and no anastomotic leaks, the gynaecologic oncology study reported lower adjusted blood loss with employed assist, and the transplant/HPB programme study reported lower elective or semi-elective conversion rates after the dedicated team intervention, with no robotic-specific complications or 30-day deaths in either era. These findings are reassuring, but should be interpreted cautiously given heterogeneous outcome definitions, limited power for uncommon adverse events, and residual confounding inherent to non-randomised designs. The absence of observed differences should not be interpreted as equivalence.

Only one included study directly examined robotic colorectal surgery, emphasising the need for colorectal-specific evaluation. Nonetheless, the mechanistic rationale for assistant model effects is relevant to pelvic colorectal workflows, where bedside tasks are central to exposure, stapling, specimen handling, and intraoperative troubleshooting. The observed reduction in non-console time in rectal resections supports prioritising complex pelvic procedures in future colorectal studies. External validity is further limited because all included studies were conducted in established United States robotic programmes, and bedside assistant roles, credentialing pathways and theatre staffing models may differ substantially across other health systems. Prospective multicentre evaluations are needed, with standardised definitions of console time, operative time, and total operating room time; explicit assistant model descriptors, including credential, stability, case volume exposure, and scope of practice; and consistent measurement of training exposure and graded complications [1, 2]. Such data are needed to inform workforce planning and robotic training pathway design.

## Conclusion

Comparative evidence directly evaluating physician versus non-physician bedside assistant models in robotic abdominal and pelvic surgery is limited to three observational studies. Available data suggest that non-physician bedside assistance may be associated with improved time-based operative efficiency in selected settings without an obvious safety penalty, although training exposure outcomes are inconsistently reported. These associations should not be interpreted as evidence of comparative effectiveness. Prospective multicentre studies using standardised definitions of operative efficiency, training exposure, and graded complications are needed, particularly in robotic colorectal surgery.

## Supplementary Information

Below is the link to the electronic supplementary material.

Supplementary Material 1

## Data Availability

No datasets were generated or analysed during the current study.
