# Supplementary material for: Bedside assistant models in robotic abdominal and pelvic surgery: a systematic review of operative efficiency, training exposure, and perioperative safety
Source: J Robot Surg. 2026 Aug 11;20(1):812. doi: 10.1007/s11701-026-03810-x (PMC13457231; doi:10.1007/s11701-026-03810-x)
Supplement: Supplementary file 1 [file 11701_2026_3810_MOESM1_ESM.docx]

**Supplementary Table 1.** Search strategy.

| **Data base** | **Search Terms** |
| --- | --- |
| Pubmed | ("Robotic"[MeSH Terms] OR "Robot"[tiab] OR "anterior resection"[tiab]) AND ("Assistance"[MeSH Terms] OR "Second surgeon"[MeSH Terms] OR "assistant"[tiab] OR "assist"[tiab] OR "bedside"[tiab]) |
| EMBASE | ('Robotic '/exp OR ' Robot ':ti,ab’) AND (' Assistance '/exp OR ‘Second surgeon'/exp OR ‘assistant':ti,ab OR ‘assist’:ti,ab OR ‘bedside':ti,ab’) |
| MEDLINE(R) and Epub Ahead of Print, In-Process & Other Non-Indexed Citations and Daily (Ovid) | (Robotic.tw OR Robot.tw) AND (Assistance.tw. OR Second surgeon.tw. OR assistant.tw. OR assist’.tw. OR bedside.tw.) |
| CENTRAL/CCTR | ("Robotic" OR "Robot”) AND ("Assistance" OR "Second surgeon" OR "assistant" OR "assist" OR "bedside") |
| Clinical trials.gov | "Robotic" OR "Robot" OR "anterior resection" AND "Assistance" OR "Second surgeon" OR "assistant" OR "assist" OR "bedside” |
| Supplementary targeted role-term search | (robot* OR robotic OR “robot-assisted” OR “robot assisted” OR “da Vinci”) AND (“physician assistant” OR “physician associate” OR PA OR “nurse practitioner” OR “advanced nurse practitioner” OR NP OR “registered nurse first assistant” OR RNFA OR “surgical care practitioner” OR SCP OR “surgical first assistant” OR “advanced practice provider” OR APP OR “employed surgical assist” OR “non-physician assistant”)  No additional eligible studies were identified. |
